# Supplementary material for: Ursolic Acid Impairs Cellular Lipid Homeostasis and Lysosomal Membrane Integrity in Breast Carcinoma Cells
Source: Cells. 2022 Dec 16;11(24):4079. doi: 10.3390/cells11244079 (PMC9776894; doi:10.3390/cells11244079)
Supplement: Supplementary file 1 [file cells-11-04079-s001.zip › supplementary materials.pdf]

A

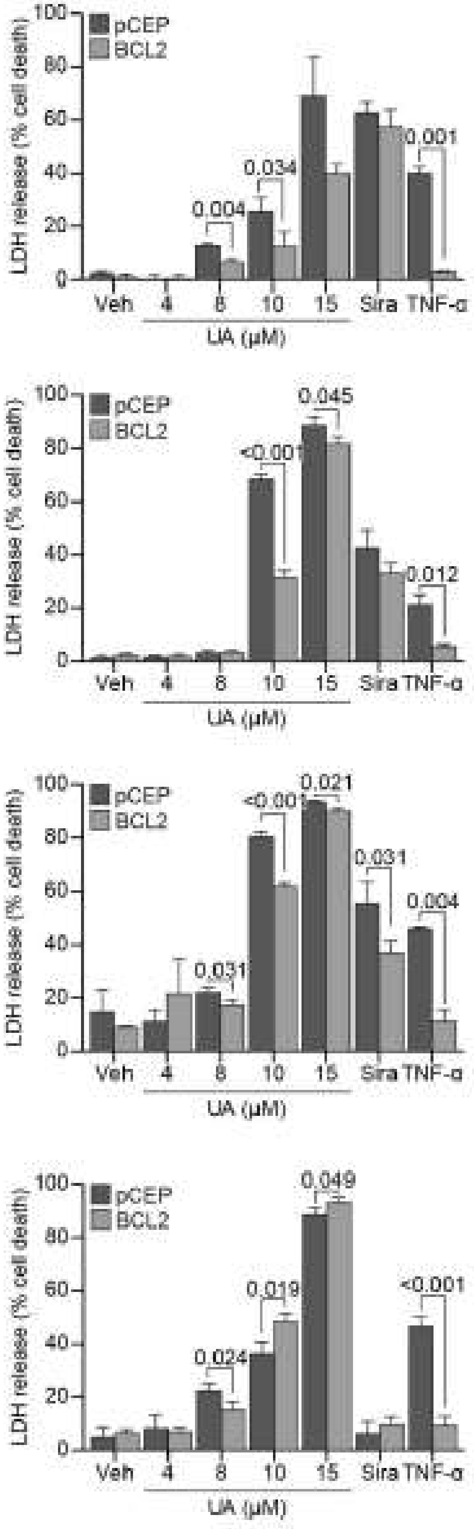

B

HCT116	Unpaired t with Welch's correction	Summary	Individual P Value
	Veh vs. UT	ns	0.8423
	Veh vs. 4	ns	0.2233
	Veh vs. 8	ns	0.4359
	Veh vs. 10	ns	0.145
	Veh vs. 12	ns	0.1918
	Veh vs. 14	ns	0.1279
	Veh vs. 16	ns	0.275
	Veh vs. 20	ns	0.1051
	Veh vs. 25	*	0.0122
	Veh vs. 30	**	0.0011
U2OS	Unpaired t with Welch's correction	Summary	Individual P Value
	Veh vs. UT	ns	0.6614
	Veh vs. 4	ns	0.3482
	Veh vs. 8	ns	0.2296
	Veh vs. 10	ns	0.2691
	Veh vs. 12	**	0.0026
	Veh vs. 14	***	0.0001
	Veh vs. 16	***	0.0001
	Veh vs. 20	****	<0.0001
HeLa	Unpaired t with Welch's correction	Summary	Individual P Value
	Veh vs. UT	ns	0.5893
	Veh vs. 4	ns	0.1982
	Veh vs. 8	ns	0.7603
	Veh vs. 10	ns	0.1029
	Veh vs. 12	ns	0.0896
	Veh vs. 16	****	<0.0001
	Veh vs. 20	**	0.0046
MCF7	Unpaired t with Welch's correction	Summary	Individual P Value
	Veh vs. UT	ns	>0.9999
	Veh vs. 4	ns	0.7743
	Veh vs. 8	ns	0.0661
	Veh vs. 10	ns	0.0992
	Veh vs. 12	*	0.0332
	Veh vs. 16	*	0.0108
	Veh vs. 20	***	0.0008
	Veh vs. 25	****	<0.0001
	Veh vs. 30	*	0.0115

Supplementary figure S1. UA kills MCF7 cells partly through apoptosis**. (A)** MCF7-*pCEP* and MCF7-*BCL2* cells were treated for 48 h with indicated concentrations of UA, 10 µM siramesine or 200 ng/ml TNF-α (top figure) or ng/ml TNF-α 20 (three bottom figures) and cell death was measured using LDH release assay. **(B)** Statistical tests of data from figure 1B. P-values were defined by multiple unpaired t-test with Welch's correction on the triplicates of each experiment comparing cell death in MCF7-*BCL2* to MCF7-*pCEP* for each treatment in (A) and comparing each treatment to vehicle in (B). Abbreviations: Same abbreviations as in figure 1.

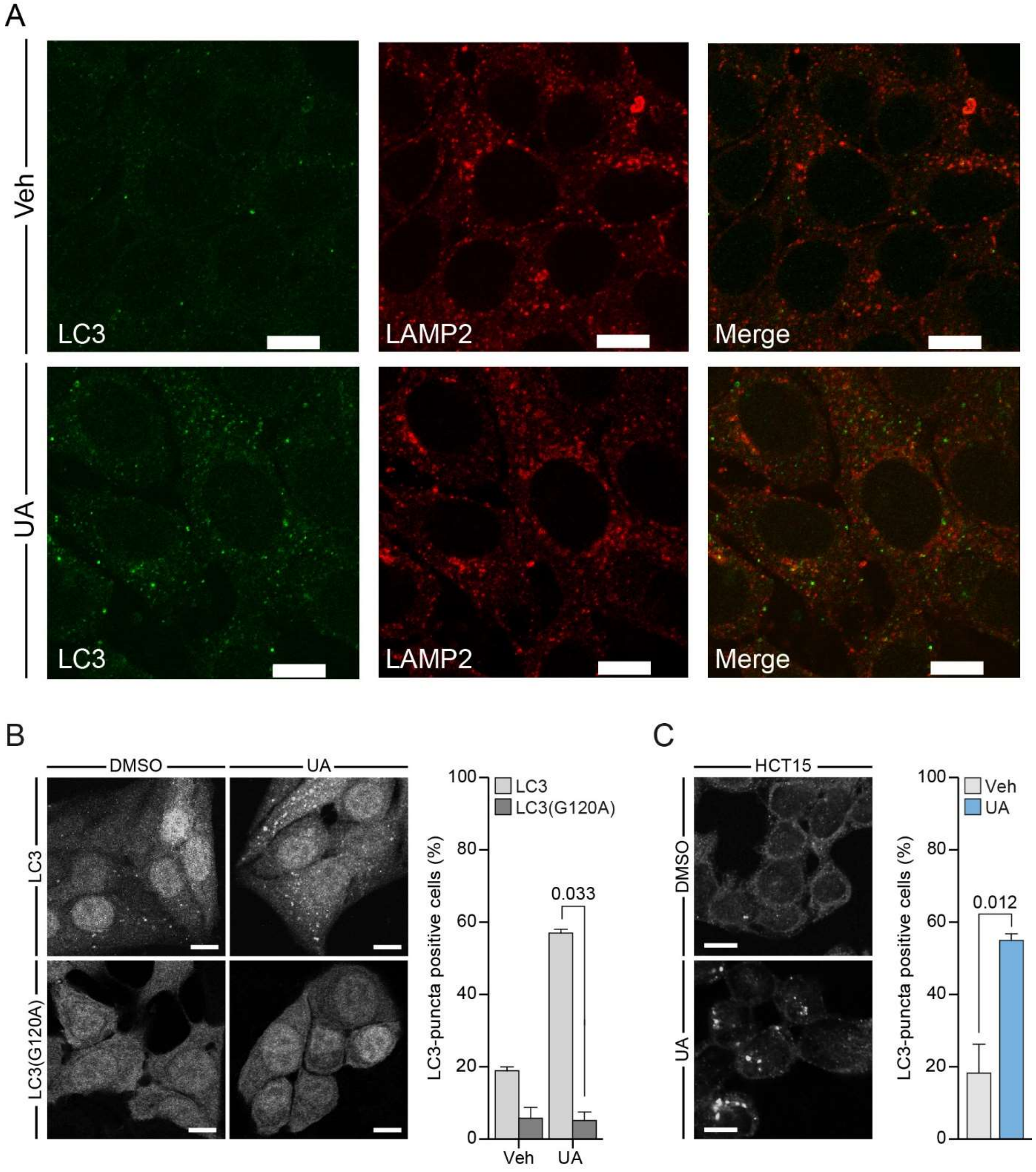

Supplementary figure S2. UA causes LC3 puncta formation in MCF7 and HCT15 cells. **(A)** MCF7 cells stained for LC3 (green) and LAMP2 (red) after treatment with vehicle (DMSO) or8 µM UA for 6 h. Bars, 10 µm **(B)** Representative images and quantification of LC3 puncta formation in MCF7 cells expressing WT GFP-LC3 or a mutant GFP-LC3(G120A) after treatment with 8 µM UA for 24 h. Bars, 10 µm. n=2 for WT GFP-LC3 and n=3 for mutant GFP-LC3(G120A) (**C)** Representative images and quantification of LC3 puncta formation in HCT15 cells after treatment with vehicle (DMSO) or 8 µM UA for 24 h. Bars, 10 µm. P-values were defined by unpaired t-test with Welch's correction comparing WT to mutant for each treatment in (B) and unpaired t-test with Welch's correction in (C). Abbreviations: (wt) wild-type, (mut) mutant. Otherwise same abbreviations as in figure 1.

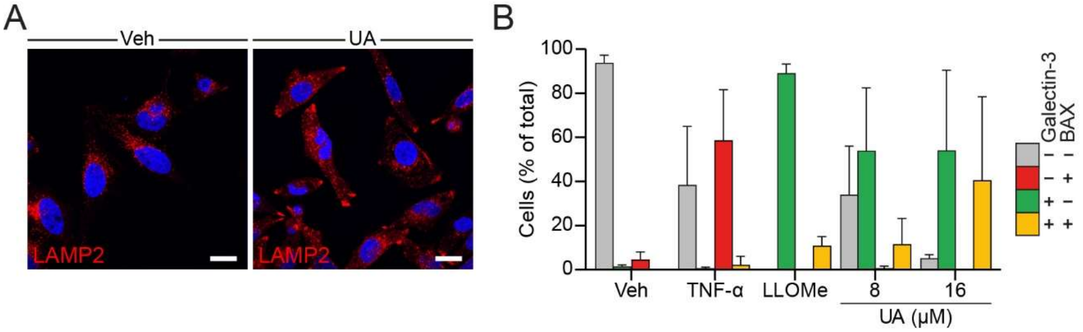

Supplementary figure S3. UA causes LMP prior to MOMP and alters lysosomal localization in HeLa cells. **(A)** LAMP2 staining in HeLa cells after 6 h vehicle (DMSO) or UA 20 μM treatment. Bars, 20 μm. (**B)** MCF7 cells were treated with 8 or 16 μM UA or 1.5 mM LLOMe for 6 h or with 20 ng/ml TNF-*α* for 24 h and co-stained for galectin-3 (green) and active BAX (red). Nuclei were labeled with Hoechst 33342 (blue). Percentages of cells with ≥ 3 galectin-3 puncta (green) or active BAX staining (red) alone or together (yellow), quantified by manual counting of 5 randomly selected fields (50-100 cells), are shown. Abbreviations: Same abbreviations as in figure 1.

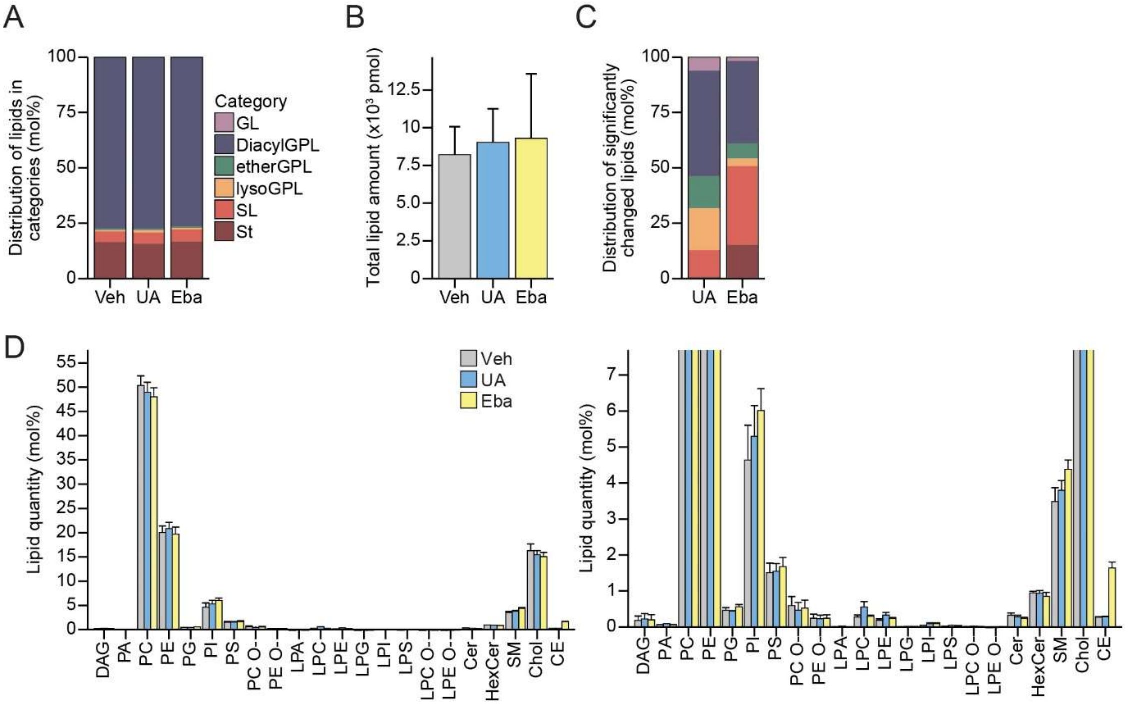

Supplementary figure S4. Additional lipidomics data. Lipidomics analysis of cells treated with vehicle (DMSO) for 8 h, 8 μM UA for 8 h or 5 μM ebastine for 6 h. **(A)** Distribution of lipids in the different lipid categories shown in mol% of total lipid (colored according to category). **(B)** Average lipid amount detected shown in pmol. **(C)** Distribution of significantly changed lipids in the different categories. Colored according to category. **(D)** Distribution of lipids in the different lipid classes shown for vehicle (DMSO), UA and ebastine treated cells. Colored according to treatment. Linear modelling with Benjamini-Hochberg correction was used to determine the significantly changed lipid species in (C). Linear modelling was performed on triplicates from three independent experiments (nine data points in total) accounting for batch factor. Abbreviations: (GL) glycerolipids, (St) sterol. Otherwise same abbreviations as in figure 1 and 5.

Table S1. List of resources

Reagent / Chemical	Source	Catalogue #; CAS #
Ammonium chloride	Sigma Aldrich	A0171; 12125-02-9
Bovine serum albumin (BSA)	VWR	422361V; 9048-46-8
ClarityTM Western ECL Substrate	Bio-Rad	170-5061; N/A (not applicable)
Concanamycin A	Santa Cruz	sc-202111; 80890-47-7
Dextran, Fluorescein and Tetramethylrhodamine, 70,000 MW, Anionic	Thermo Fisher Scientific	D1951; N/A
Digitonin	Sigma Aldrich	D141; 11024-24-1
DMSO	VWR	VWRCN182, 67-68-5
Ebastine	Cayman Chemical	15372, 90729-43-4
EnduRen™	Promega	E6481; N/A
Geneticin (G418 Sulfate)	Life Technologies	11811-031, 108321-42-2
Goat serum	DAKO	X0907; N/A
Hoechst 33342	Sigma Aldrich	B2261; 23491-52-3
Live Cell Imaging Solution	Thermo Fisher Scientific	A14291DJ; N/A
LLOMe	Santa Cruz	SC-285992, 16889-14-8
Methanol	VWR	34966; 67-56-1
MiniProtean TGX gels, 15-well, 4-15%	Bio-Rad	456-1086; N/A
Nitrocellulose membrane	Bio-Rad	170-4158; N/A
Novex™ Sharp Pre-stained Protein Standard	Thermo Fisher Scientific	LC5800; N/A
Nuclear Violet	ATT Bioquest	17543; N/A
Paraformaldehyde (PFA) in PBS	Ampliqon	432.261.000, 30525-89
Pierce™ BCA assay kit	Thermo Fisher Scientific	#23225;N/A
Prolong Gold Antifade mounting medium	Life Technologies	P36930; N/A
Propidium Iodide	Sigma Aldrich	P4864; 25535-16-4
Rapamycin	Sigma Aldrich	R0395; 53123-88-9
Rapamycin	Sigma Aldrich	553210; 53123-88-9
SiR-tubulin	Spirochrome	SC002; N/A
Siramesine	Gift from Christine Volbracht, H. Lundbeck A/S	N/A, 163630-79-3
SuperSignal West Femto Maximum Sensitivity Substrate	Thermo Fisher Scientific	PI34096, N/A
TNF alpha (human)	Sigma Aldrich	T0157; 94948-59-1
TNF alpha (human)	Sigma Aldrich	H8916; 94948-59-1
Triton-X-100	Sigma Aldrich	T9284; 9002-93-1
Tween-20	VWR	A4974.0500; 9005-64-5
Ursolic Acid	Abcam	ab141113; 77-52-1
**Antibodies**	**Source**	**Identifier**
Active Bax	Cell Signaling Technology	2772
GFP	Abcam	ab290

Cathepsin B	Gift from Ekkehard Weber	https://www.ncbi.nlm.nih.gov/pmc/articles/PMC4496956/#!po=35.0000
Cathepsin B	Upstate	06-480
EEA1	Abcam	ab2900
Galectin 3	Sigma Aldrich	MABT51
HSP90	BD Transduction Laboratories 6	610418
LAMP2	Developmental Studies Hybridoma Bank	H4B4
MAP1LC3	Cell Signaling Technology	3868
Mouse IgG (donkey) Alexa Fluor 488	Thermo Fisher Scientific	A-21202
Mouse IgG (donkey) Alexa Fluor 594	Thermo Fisher Scientific	A21203
Mouse IgG (donkey) Alexa Fluor 647	Thermo Fisher Scientific	A31571
Mouse IgG (rabbit) HRP-conjugated	Sigma Aldrich	A9044-2ML
Rabbit IgG (donkey) Alexa Fluor 488	Thermo Fisher Scientific	A-21206
Rabbit IgG (donkey) Alexa Fluor 568	Thermo Fisher Scientific	A10042
Rabbit IgG (donkey) Alexa Fluor 594	Thermo Fisher Scientific	A-21207
Rabbit IgG (goat) HRP-conjugated	Vector Laboratories	PI-1000
Total alpha tubulin	Abcam	ab15246
MAP1LC3B	Nanotools	Clone
KITS	Source	Identifier
LDH release Assay	Roche	11644793001
Pierce BCA Protein Assay Kit	Thermo Fisher Scientific	23225
Software	Source	Identifier
ImageJ	Fiji	www.imagej.net/Fiji
LipidXplorer		[44]
MetaXpress version 6.6.1.42	Molecular Devices	
Prism 9	GraphPad	https://www.graphpad.com/scientific-software/prism/
R version 4.0.5	R core team (2021)	https://www.R-project.org/
Rstudio version 4.0.1717	Rstudio team 2021	http://www.rstudio.com/
ScanR Acquisition software 3.1	Olympus	https://www.olympus-lifescience.com/en/microscopes/inverted/scanr/#!cms[focus]=cmsContent6166
ScanR Analysis Software 3.1	Olympus	https://www.olympus-lifescience.com/en/microscopes/inverted/scanr/#!cms[focus]=cmsContent6166
TIBCO Spotfire	TIBCO Software Inc.	https://www.tibco.com/products/tibco-spotfire
Xcalibur	Thermo Scientific	OPTON-30965

Zen	Zeiss	https://www.zeiss.com/microscopy/int/products/microscope-software/zen.html
Cell culture	Source	Identifier
Dulbecco's phosphate-buffered saline (DPBS) w/o Ca2+/Mg2+	Thermo Fisher Scientific	14190-094
Dulbecco's phosphate-buffered saline (DPBS) with Ca2+/Mg2+	Thermo Fisher Scientific	14040-117
Dulbecco's Modified Eagle's medium (DMEM) + GlutaMAX	Thermo Fisher Scientific	31966-021
Fetal calf serum	Thermo Fisher Scientific	10270-106
GlutaMax Supplement	Thermo Fisher Scientific	35050-061
HCT116	A gift from S. Shirasawa	[38]
HCT15, colorectal adenocarcinoma	ATCC	ATCC® CCL-225™
HeLa	ATCC	ATCC® CCL-2™
MCF7-BCL-2	Marja Jäättelä, Danish Cancer Society Research Center	[34]
MCF7-pCEP	Marja Jäättelä, Danish Cancer Society Research Center	[34]
MCF7-S1, mammary adenocarcinoma (female)	Marja Jäättelä, Danish Cancer Society Research Center	PubMed PMID: 7540278
Minimum Essential Medium (MEM) Non-Essential Amino Acids (NEAA)	Thermo Fisher Scientific	11140035
Penicillin/Streptomycin	Life Technologies	11811-031
RPMI 1640 Medium + GlutaMax, phenol red	Thermo Fisher Scientific	61870
RPMI 1640 Medium, no glutamine, no phenol red	Thermo Fisher Scientific	32404014
U-2-OS human osteosarcoma	ATCC	ATCC® HTB-96™
MCF7-eGFP-LC3wt	Marja Jäättelä, Danish Cancer Society Research Center	[34]
MCF7-eGFP-LC3(G120A)	Marja Jäättelä, Danish Cancer Society Research Center	[34]
MCF7-tfLC3	Marja Jäättelä, Danish Cancer Society Research Center	[35]
MCF-7-RLuc-LC3wt	Marja Jäättelä, Danish Cancer Society Research Center	[36]
MCF-7-RLuc-LC3(G120A)	Marja Jäättelä, Danish Cancer Society Research Center	[36]
Plates	Source	Identifier
Screenstar 96-well	Greinar Bio One	655866

**Table S2:** Internal lipid and drug standards				
**Lipid class**	**Sum formula**	**Source**	**ID**	**Amount added (pmol)**
DAG	DAG 12:0/12:0	Avanti	800812	8
PA and PA O-	PA 12:0/12:0	Avanti	840635	16.34
PC and PC O-	PC 12:0/12:0	Avanti	850335	20
PE and PE O-	PE 12:0/12:0	Avanti	850702	20
PG and PG O-	PG 12:0/12:0	Avanti	840435	11.03
BMP	BMP 14:0/14:0	Avanti	110857	12
PI and PI O-	PI 8:0/8:0	Avanti	850181	10.89
PS and PS O-	PS 12:0/12:0	Avanti	840038	6.63
LPA and LPA O-	LPA 17:0	Avanti	11067	17.64
LPC and LPC O-	LPC 12:0	Avanti	855475	16
LPE and LPE O-	LPE 13:0	Avanti	110696	17.36
LPG and LPG O-	LPG 17:1	Avanti	858127	10.46
LPI and LPI O-	LPI 13:0	Avanti	110716	11.28
LPS and LPS O-	LPS 17:1	Avanti	858141	14.72
Ceramide (Cer)	Cer 18:1;2/12:0;0	Avanti	860512	16
HexCer	HexCer 18:1;2/12:0;0	Avanti	860543	20
SM	SM 18:1;2/12:0;0	Avanti	860583	13.62
Cholesterol	Chol-D4	QMX	D-6359	196.25
CE	CE-D7 15:0	Avanti	700144	17.78
Ebastine	Ebastine-D5	Toronto research chemicals	E320002	2
Carebastine	Carebastine-D5	Toronto research chemicals	C183442	2

Table S3: Precursor ion, fragment ion, and neutral loss for lipid identification

Lipid class	Mode	MS1: Precursor ion	MS2: Fragment ion	MS2: Neutral loss	MS2: *m/z*	MS2: Species specifics
DAG	POS	$[M+NH_4]^+$		[Fatty acid – H + NH_4]		All
PA, PA O-, LPA	NEG	$[M–H]^-$	[Glycerophosphate – H – $H_2O]^-$		152.9958	All
		$[M–H]^-$	[Fatty acid – $H]^-$		*	PA, PA O-, LPA
PA, PA O-, LPA<br>PC, PC O-, SM, LSM	NEG<br>POS	$[M–H]^-$	[Fatty acid O- – $H]^-$		*	PA O-
		$[M+H]^+$	[Phosphorylcholine + $H]^+$		184.0733	All
LPC, LPC O-	NEG	$[M–H]^-$	[Fatty acid – $H]^-$		*	LPC
PE, PE O-, LPE, LPE O-	NEG	$[M–H]^-$	[Ethanolaminephosphate – H – $H_2O]^-$		196.038	All
		$[M–H]^-$	[Fatty acid – $H]^-$		*	PE, PE O-, LPE
PE, PE O-, LPE, LPE O-<br>BMP/PG, LPG	NEG<br>NEG	$[M–H]^-$	[Fatty acid O- – $H]^-$		*	PE O-
		$[M–H]^-$	[Glycerophosphate – H – $H_2O]^-$		152.9958	All
BMP/PG, LPG<br>PI, PI O-, LPI, LPI O-	NEG<br>NEG	$[M–H]^-$	[Fatty acid – $H]^-$		*	BMP/PG, LPG
		$[M–H]^-$	[Glycerophosphate – H – $H_2O]^-$		152.9958	All
		$[M–H]^-$	[Inositolphosphate – H – $H_2O]^-$		241.0119	All
PI, PI O-, LPI, LPI O-<br>PS, PS O–, LPS	NEG<br>NEG	$[M–H]^-$	[Fatty acid – $H]^-$		*	PI, PI O-, LPI
		$[M–H]^-$	[Fatty acid O- – $H]^-$		*	PI O-
		$[M–H]^-$	[Glycerophosphate – H – $H_2O]^-$		152.9958	All
		$[M–H]^-$		[$C_3H_5NO_2$]<br>Δ*m/z*: 87.032		All
PS, PS O–, LPS, LPS O–	NEG<br>NEG	$[M–H]^-$	[Fatty acid – $H]^-$		*	PS, PS O-, LPS
		$[M–H]^-$	[Fatty acid O- – $H]^-$		*	PS O-
		$[M1–2H]^{2+}$	[Fatty acid – $H]^-$		*	All

Cer, HexCer,	POS	$[M+H]^+$	$[LCB + H - H_2O]^+$	*	All
Cer, HexCer,	POS	$[M+H]^+$	$[LCB + H - 2H_2O]^+$	*	All
	POS	$[M+H]^+$	$[LCB + H - 2H_2O]^+$	*	All
CE	POS	$[M+NH_4]^+$	$[Chol - NH_3 - H_2O]^+$	369.3516	All
Chol	POS (SIM/ tPRM)	$[M+NH_4]^+$	$[Chol - NH_3 - H_2O]^+$	369.3516	All
